# A New Ala-122-Asn Amino Acid Change Confers Decreased Fitness to ALS-Resistant *Echinochloa crus-galli*

**DOI:** 10.3389/fpls.2017.02042

**Published:** 2017-11-28

**Authors:** Silvia Panozzo, Laura Scarabel, Valentina Rosan, Maurizio Sattin

**Affiliations:** Institute of Agro-environmental and Forest Biology – Consiglio Nazionale delle Ricerche, Padua, Italy

**Keywords:** barnyardgrass, phenology, biomass allocation, target-site resistance, Ala-122-Asn mutation, seed production, herbicide resistance management

## Abstract

Gene mutations conferring herbicide resistance may cause pleiotropic effects on plant fitness. Knowledge of these effects is important for managing the evolution of herbicide-resistant weeds. An *Echinochloa crus-galli* population resistant to acetolactate synthase (ALS) herbicides was collected in a maize field in north-eastern Italy and the cross-resistance pattern, resistance mechanism and fitness costs associated to mutant-resistant plants under field conditions in the presence or absence of intra-specific competition were determined. The study reports for the first time the Ala-122-Asn amino-acid change in the ALS gene that confers high levels of cross-resistance to all ALS inhibitors tested. Results of 3-year growth analysis showed that mutant resistant *E. crus-galli* plants had a delayed development in comparison with susceptible plants and this was registered in both competitive (3, 7, and 20 plants m^-2^) and non-competitive (spaced plants) situations. The number of panicles produced by resistant plants was also lower (about 40% fewer panicles) than susceptible plants under no-intraspecific competition. Instead, with the increasing competition level, the difference in panicle production at harvest time decreased until it became negligible at 20 plants m^-2^. Evaluation of total dry biomass as well as biomass allocation in vegetative parts did not highlight any difference between resistant and susceptible plants. Instead, panicle dry weight was higher in susceptible plants indicating that they allocated more biomass than resistant ones to the reproductive organs, especially in no-competition and in competition situations at lower plant densities. The different fitness between resistant and susceptible phenotypes suggests that keeping the infestation density as low as possible can increase the reproduction success of the susceptible phenotype and therefore contribute to lowering the ratio between resistant and susceptible alleles. If adequately embedded in a medium or long-term integrated weed management strategy, the presence of R plants with a fitness penalty provides an opportunity to minimize or reverse herbicide resistance evolution through the implementation of integrated weed management, i.e., all possible control tools available.

## Introduction

Weed resistance to herbicides is a severe threat to the sustainability of intensive cropping systems. Herbicides applied to large weed populations impose a strong selection pressure that has led to the evolution of many resistant populations worldwide (Heap, 2017). Weeds can withstand herbicide effects because of the presence of mutated resistant alleles (Powles and Yu, 2010). Target site resistance mechanism (TSR) is determined by mutations causing structural changes at the herbicide binding site, therefore limiting the herbicide impact. Instead non-target site resistance (NTSR) includes all mechanisms able to reduce the quantity of herbicide that reaches the target-site. These mechanisms can be determined by modifications at the active site of a metabolic enzyme or at a transporter protein that enhances these proteins’ activity in herbicide degradation or compartmentation/sequestration away from its site of action (Yuan et al., 2007; Powles and Yu, 2010).

Theory predicts that any mutation endowing herbicide resistance may be associated with negative pleiotropic effects on fitness, also known as adaptation cost (Purrington, 2000; Vila-Aiub et al., 2009) and defined as the reduction of plant fitness in a herbicide-free environment caused by negative pleiotropic effects of resistance alleles (Vila-Aiub et al., 2015).

Plant relative fitness, which according to Primack and Kang (1989) is defined as the average success in producing offspring contributing to the next generation by a particular phenotype relative to another phenotype (i.e., resistant vs. susceptible in the case of resistance costs), plays an important role in evolutionary terms. Fitness is a phenotypic response that depends on the evolved life-history traits, and therefore, it is greatly affected by environmental conditions and genetic variation (Primack and Kang, 1989). In particular, resistance costs expression has been shown to depend on several factors: resistance mechanism involved (Vila-Aiub et al., 2005a), specific mutant resistance allele (Menchari et al., 2008), dominance of the resistance cost (Roux et al., 2004), pleiotropic effects on the kinetics of herbicide target proteins (Yu et al., 2010), genetic background (Paris et al., 2008), and environmental conditions (Gassmann, 2005).

The resistance cost is estimated by determining the difference in fitness between a herbicide-resistant (R) and herbicide-susceptible (S) genotype. In this context, it is important that individuals share a similar genetic background, except for the alleles endowing herbicide resistance (Vila-Aiub et al., 2009). In this way, the genetic variability between R and S genotypes is limited and only fitness costs associated to those genes that endow resistance are assessed (Vila-Aiub et al., 2011).

A better understanding of the cost/benefit trade-offs of resistant and susceptible alleles is crucial for predicting the herbicide resistance evolution, and more generally the dynamics of weed populations (Menalled et al., 2016). In the last three decades, the widespread and rapid evolution of herbicide resistance produces evidence that resistance mutations confer large fitness benefits in the presence of herbicides, however, several studies suggest that resistance costs when herbicides are not applied range from moderate to relatively small (Vila-Aiub et al., 2009). In an agricultural environment, where herbicides may be rotated or mixed and/or used less frequently, resistance will evolve where the fitness advantage in the presence of certain herbicides (resistance benefit) is greater than the resistance cost (Neve et al., 2014). In other words, resistance costs will affect resistance evolution in environments when the selecting agent(s), and possibly other actives with the same site of action, are less or no longer used. From a management perspective, a better insight into the life history trade-offs associated with resistance may have practical implications in devising strategies which manipulate fitness costs that can result in selection against resistance alleles.

*Echinochloa crus-galli* (L.) P. Beauv. (barnyardgrass) is a polyploid, therophyte, predominantly self-pollinating, summer annual weed that grows well in organic soils. It has a C_4_ photosynthetic cycle that contributes to making it a very successful competitor and one of the most problematic weeds in maize, rice, and other summer crops. It is a prolific seed producer, which often results in considerable soil seed banks (Holm et al., 1977). A healthy plant can yield up to more than 400,000 seeds (Norris, 1992), making it very problematic for crop production if not adequately controlled. *E. crus-galli* is ranked second globally as a weed that evolved resistance to numerous herbicides with different sites of action (Heap, 2017). Since 2000, resistance to herbicides inhibiting acetolactate synthase (ALS; also referred to as acetohydroxyacid synthase, AHAS; EC 2.2.1.6), a key enzyme in the synthesis of branched chain amino acids, has been reported in several European countries, i.e., Italy, Spain, Austria, France, and Germany (Heap, 2017).

Acetolactate synthase inhibitors are the herbicides most prone to select for resistance in weeds (Beckie and Tardif, 2012; Yu and Powles, 2014). In many cases, evolved resistance is target-site-based, caused by amino acid substitutions in one of the conserved regions of the ALS target enzyme. Over the past 27 years, 29 resistance-endowing amino acid substitutions at eight positions of the AHAS gene in 64 weed species have been identified (reviewed by Tranel et al., 2017).

Resistance costs associated with ALS resistance have been studied in a limited number of cases and the costs often were incorrectly determined because of inappropriate plant material, lack of identification of the resistance mechanism involved or inappropriate methodology to measure fitness (Vila-Aiub et al., 2015). Fitness consequences have been assessed for the Pro-197-His substitution in *Lactuca serriola* L., where a reduction in vegetative biomass of resistant compared with susceptible plants growing under competitive conditions has been demonstrated (Alcocer-Ruthling et al., 1992). Substantial pleiotropic effects on plant morphology and anatomy, causing a fitness cost, have been described in field evolved ALS-resistant *Amaranthus powellii* S. Watson with the Trp-574-Leu ALS mutation (Tardif et al., 2006). More recent studies, where ALS resistance alleles were identified and genetic background of resistant and susceptible plants was adequately considered, have assessed the subtle impact of ALS resistance on plant growth and fitness. In homozygous *Lolium rigidum* Gaudin plants with an ALS allele endowing resistance (Ala-197, Arg-197, Glu-197, Ser-197, or Leu-574), no pleiotropic effects on vegetative growth were found (Yu et al., 2010). Similarly, it was demonstrated that the ALS alleles Tyr-122, Ser-197, Glu-376, and Leu-574 do not cause negative pleiotropic effects on *Raphanus raphanistrum* L. vegetative growth (Li et al., 2013). All these findings highlight that the impact of resistance-endowing ALS gene mutations on plant fitness is dependent on weed species and mutant resistant ALS allele considered. Therefore, generalization is impossible.

In this research the so-called ‘single-population’ approach (Vila-Aiub et al., 2005b) was used: two seed stocks, S and R, were preliminarily selected from plants harvested in the same site highly infested by *E. crus-galli* resistant to ALS inhibitors. The aims of the research were: (1) to confirm the resistance to ALS-inhibiting herbicides and determine the cross-resistance pattern of the R sub-population; (2) to determine the resistance mechanism(s) involved; (3) to assess the fitness costs associated to mutant ALS-resistant *E. crus-galli* plants under field conditions, in the presence or absence of intra-specific competition.

## Materials and Methods

### Plant Material

#### Experimental Maize Field

The experimental field site was located in Cona (Venice province, Veneto region, NE Italy, N 45° 09’ 27.2”, E 12° 09’ 12.7”, -1.6 m below sea level) where continuous silage maize had been cultivated since the end of the 1950s. No sprinkler irrigation is used in the area and soil moisture is maintained by keeping a high level of water in the ditches. The soil is highly organic (around 27% C), not allowing the application of pre-emergence herbicides and so chemical weed control is only done in post-emergence, mainly with ALS inhibitors. Since 2002 the control of *E. crus-galli* by ALS inhibitors has become progressively unsatisfactory and in 2005, seeds were collected from about 40 plants that survived the sulfonylurea treatments (i.e., two treatments with nicosulfuron 60 g a.i. ha^-1^) (population 05-31).

Greenhouse bioassay conducted by the Italian Herbicide Resistance Working Group (GIRE, 2017) confirmed that population 05-31 was highly resistant to the ALS inhibitor nicosulfuron, while it was controlled by all other herbicides with a different site of action (i.e., terbuthylazine+mesotrione, fluazifop and *S*-metolachlor). A population is defined as highly resistant to a herbicide when plant survival is >20% at the recommended field dose (1x) as well as >10% at dose 3x (Panozzo et al., 2015). For population 05-31, plant survival of nicosulfuron was not dose-dependent (69% ± 2.4 at dose 1x and 73% ± 15.8 at dose 3x) and the population was ascribed as highly resistant.

#### Selection of Controlled Genetic Background Sub-populations

In 2011, 100 *E. crus-galli* seedlings at two to three leaf stages were harvested in the field, transplanted into pots and grown to the stage of three to four tillers in the greenhouse. Two vegetative clones from each individual plant were then produced: two tillers with intact roots were excised from each parent plant, trimmed to 2 cm of shoot material and individually repotted. Five days later, one clone for each parent plant was sprayed with nicosulfuron (60 g a.i. ha^-1^). Four weeks after treatment (WAT) resistant (R) and susceptible (S) plants were assessed and the phenotype of the respective untreated clone was identified. Then, 30 untreated R and S plants were placed in the greenhouse in separate compartments. Even if *E. crus-galli* is predominantly a self-pollinated species, S plants were kept separated from the R ones to avoid any possible cross pollination. Mature seeds were collected from senescent plants, cleaned and stored at 20°C. The bulk of S and R plants (hereafter called simply sub-populations S and R), were used to determine the cross-resistance pattern, the resistance mechanism(s) involved and ultimately for the in-field fitness experiments.

### Resistance Status and Resistance Mechanisms Involved

#### Dose–Response Pot Experiment

An outdoor dose–response experiment was conducted to determine the level of resistance of the S and R sub-populations of *E. crus-galli* to nicosulfuron (sulfonylurea), penoxsulam (triazolopyrimidine), bispyribac-Na (pyrimidinylthiobenzoate), and imazamox (imidazolinone), four ALS herbicides belonging to different chemical families (**Table 1**). Seeds were chemically scarified in concentrated sulfuric acid (96%) for 20 min, rinsed with water and sown in plastic boxes (10 cm × 10 cm × 6 cm) containing peat. Boxes were then placed in a germination cabinet for 6 days at 15/25°C (night/day) and 12 h photoperiod with neon tubes providing a Photosynthetic Photon Flux Density (PPFD) of 15–30 μmol m^-2^ s^-1^. Twenty seedlings, at one-leaf stage (BBCH 11 – Hess et al., 1997), were transplanted into plastic trays (325 mm × 265 mm 95 mm) with a standard potting mix (60% silty loam soil, 15% sand, 15% perlite, and 10% peat). The trays were placed in a greenhouse where temperature ranged from 15 to 19°C and from 26 to 33°C night/day, respectively, and watered daily to maintain the substrate at or near field capacity. When plants reached the two to three leaf stages (BBCH 12-13), herbicides were applied as commercial formulations (**Table 1**), with recommended surfactants, using a precision bench sprayer delivering 300 L ha^-1^, at a pressure of 215 kPa and a speed of 0.75 m s^-1^, with a boom equipped with three flat-fan (extended range) hydraulic nozzles (TeeJet^®^, 11002). Each sub-population was treated with seven doses of each herbicide calculated following a geometric progression and ranging from 1/16x to 2x (plus the dose 2/3x) and from 1/4x to 16x for S and R sub-populations, respectively. The experimental design was a complete randomized block with three replicates (i.e., one tray per replicate) per herbicide dose.

**Table 1:** Details of herbicides used in the dose–response experiment.

Active ingredient (a.i.)	Commercial product	Company	Concentration (g a.i. L^-1^)	Field dose (1x) (L ha^-1^)
Nicosulfuron	Ghibli	Syngenta	40	1.5
Penoxsulam	Viper	Dow Agrosciences	22.5	2.7
Bispyribac-Na	Nominee^∗^	Bayer CropScience	408	0.075
Imazamox	Altorex^∗∗^	BASF	40	1

*^∗^ Applied with Biopower 1 L ha^-*1*^. ^∗∗^ Applied with Dash 0.5%.*

Plant survival and shoot fresh weight per tray were recorded four WAT and expressed as percentage of the untreated control. Dead plants or plants with dead leaves and no regrowth from the basal part of the plant were classified as susceptible. Plant survival and fresh weight was expressed as percentage of untreated control and standard error was calculated per each mean value.

#### Detection of Mutation in ALS Gene

To check for mutation(s) endowing herbicide resistance, 10 plants from sub-population S and 10 plants from sub-population R were analyzed. Total RNA was extracted from 100 mg of young leaf tissue (one leaf per plant) from plants that survived a treatment with nicosulfuron at dose 60 g a.i. ha^-1^ using the Invitrap^®^ spin plant RNA mini kit (Stratec Biomedical AG, Germany). Nucleic acid concentration was measured using a NanoDrop 2000c Spectrophotometer (NanoDrop Products, United States). cDNA was synthesized using the ImProm-II^TM^ Reverse Transcription System (Promega, United States) as follows: 1 μg of target RNA and 0.5 μg of oligo dT_15_ were mixed with nuclease free water to a final volume of 5 μL; samples were incubated for 5 min at 70°C and then quick-chilled for 5 min on ice; after this denaturation step, the reaction mix (2.25 mM MgCl_2_, 0.5 mM dNTP mix, 1 μL of Improm-II^TM^ Reverse Transcriptase, 1x concentration of supplied buffer and nuclease free water to a final volume of 15 μL) was added to each sample and samples were then placed in a T1 plus Thermocycler 96 (Biometra, Germany) at 25°C for 5 min, 1 h at 42°C, and 10 min at 70°C.

The pair of primers ECH-ALS-F (5′- TCGCAAGGGCGCGGACATCCTCGT -3′) and ECH-3R (5′- TCCTGCCATCACCHTCCAKGA -3′) were used to amplify the full ALS gene sequence including all known conserved domains (Panozzo et al., 2013). PCR amplification was conducted using the Advantage^®^ 2 PCR kit (Clontech) in a 50 μL mixture of 1x Advantage^®^ 2 SA PCR Buffer, 1x dNTP mix, 0.2 μM of each primer, 5% DMSO, 1x Advantage^®^ 2 Polymerase Mix and 100 ng cDNA. Amplification was conducted using the following program: 1 min at 95°C; 35 cycles of 30 s at 95°C, 30 s at 60°C, and 120 s at 68°C; 3 min at 68°C. PCR products were purified with NucleoSpin^®^ Gel and PCR Clean-up kit (Macherey-Nagel GmbH & Co., Germany), sequenced by BMR Genomics (Padova, Italy) and edited with FinchTV 1.4.0.

Once the full ALS length had been sequenced and mutation endowing resistance identified, only a 500 bp amplicon starting from the 5′-end part of the ALS gene was amplified for all R and S plants in order to individuate the amino-acid at position 122 of the ALS gene (position referred to the ALS sequence of *Arabidopsis thaliana*). The PCR amplification was conducted as described above with primer ECH-ALS-F and the reverse primer ECH-5RACE-1 (5′-GCCGCGACTCACCAACAAGA-3′) and with the following Thermocycler program: 1 min at 95°C; 35 cycles of 30 s at 95°C, 30 s at 58°C, and 40 s at 68°C; 3 min at 68°C.

#### ALS Enzyme *in Vitro* Bioassay

ALS enzyme bioassay was performed to measure the activity of the enzyme exposed to increasing doses of herbicides in the two sub-populations S and R.

Proteins were extracted from seedlings fresh leaf tissue as described by Wright et al. (1998) with slight modifications. Five grams of the youngest leaves, pooled from 40 to 50 plants, were homogenized with liquid nitrogen in a mortar and suspended in 35 mL of ice-cold homogenization buffer [100 mM potassium phosphate buffer (pH 7.2), 5 mM Na-pyruvate, 5 mM MgCl_2_, 10 μM flavin adenine dinucleotide (FAD), 1 mM thiamine pyrophosphate (TPP), 1 mM dithiothreitol (DTT), 10% (V/V) glycerol] and 1% (wt/wt) polyvinylpolypyrrolidone (PVPP). The homogenate was filtered through four layers of cheesecloth, and then centrifuged at 20,000 × *g* for 20 min at 4°C. The supernatant was decanted to a new centrifuge tube and an appropriate quantity of ammonium sulfate was added to create a 45% (wt/V) solution. The mixture was stirred gently for 1 h on ice, and then centrifuged at 20,000 × *g* for 20 min at 4°C. The resultant pellet was dissolved in 3 mL of re-suspension buffer [50 mM potassium phosphate buffer (pH 7.2), 5 mM MgCl_2_, 1 mM TPP, and 10 μM FAD]. The resulting solution was desalted using Disposable PD-10 Desalting Columns (GE Healthcare). Protein concentration of the extract was determined using a NanoDrop 2000c Spectrophotometer.

ALS enzyme assays were performed as described by Schmitzer et al. (1993). Enzyme activity was determined based on the amount of acetoin formed from acetolactate using the method of Westerfeld (1945). Each reaction contained 50 μL of protein extract (2.0 μg μL^-1^), 50 μL of the standard reaction buffer [50 mM potassium phosphate buffer (pH 7.0), 100 mM Na-pyruvate, 5 mM MgCl_2_, 1 mM TPP, 10 μM FAD] and 50 μL of various concentrations of the technical grade of imazamox and nicosulfuron. A positive control without herbicide and a blank with sulfuric acid (see below) were included. The two sub-populations were assayed with two ALS inhibitors, the selecting agent nicosulfuron and imazamox, the ALS inhibitor that had given the highest RI in the whole-plant pot bioassay. The experiment was repeated twice, with three replicates per herbicide concentration. Herbicide concentrations ranged from 10^-3^ to 10^6^ nM in 10-fold increments. The mixtures were incubated at 37°C for 90 min. The reactions were stopped by adding 25 μL of 3.5% (V/V) sulfuric acid, and then incubated at 60°C for 20 min. The amount of acetoin formed was determined by incubating the mixture with 150 μL of 0.55% (wt/V) creatine, 5.5% (wt/V) α-naphthol, and 1.375 N NaOH at 37°C for 40 min. Acetoin concentration was measured by spectrophotometry at a wavelength of 530 nm.

The R/S ratio was obtained as the ratio between the *I*_50_ of sub-populations R and S.

### Growth Analysis in Field Experiments in Different Competitive Situations

To detect if a fitness cost was associated with the resistant allele, a 3-year field comparative growth analysis was conducted in north-eastern Italy in 2012 (experiment 1), 2013 (experiment 2), and 2014 (experiment 3) on the farm where the original resistant population 05-31 had been collected. In each experiment *E. crus-galli* plants growth were followed from the beginning of May to mid/end of September (i.e., to maize harvest). R and S plants were grown in virtually non-competitive (hereafter called no-competition) and intraspecific competitive situations: well-spaced (plants were 2 m apart, no-competition) as well as at 3, 7, and 20 plants m^-2^ (competition). In competitive situations, each target plant was surrounded by 12 equally spaced plants of the other phenotype (i.e., the S target plant was surrounded by R plants and vice-versa) (Beruto et al., 1996). The experimental design was a completely randomized block with three replicates (see Supplementary Figure S1).

At the beginning of May, seeds of the two sub-populations S and R were chemically scarified in concentrated sulfuric acid (96%) for 20 min, carefully rinsed and conserved in a solution of KNO_3_ at 2% (wt/V) until sowing. Sowing was done in the field by putting a few seeds in equidistant pockets (dibbling method). To make sure that the germinated plants were of the correct phenotype, some sterilized soil was put in a buried plastic tube (10 cm × 10 cm) and 15 seeds were sown inside and watered daily. Then seedlings were thinned to one plant and plastic tubes were removed to allow the plants to grow freely.

Phenology of the two phenotypes was monitored throughout the growing cycle: beginning of flowering was recorded for the target plants in all treatments; panicle numbers were counted weekly and covered with glassine bags (Glassine Bag Co. Global Polythene, Corringham, Essex, United Kingdom) just prior to the onset of seed shattering.

Temperature sum was calculated based on Growing-Degree Day equation (GDD):

$$\text{GDD}\;\text{=}\;[(T_{\text{max}}\;\text{-}\;T_{\text{min}})\text{/2}]\;\text{-}\;T_{\text{b}}$$

where *T*_max_ and *T*_min_ are the daily maximum and minimum temperatures, respectively, and *T*_b_ is the base temperature, estimated to be 10.5°C for *E. crus-galli* (Sartorato and Pignata, 2008). When *T*_min_ was ≤T_b_, the value of *T*_b_ was considered. Air temperature and rainfall data were recorded by the nearest ARPAV (Agenzia Regionale per la Prevenzione e Protezione Ambientale del Veneto^1^) weather station (about 8 km away from the experimental field).

In 2012 and 2013 other three well-spaced plants for each phenotype were grown and panicles of these plants were covered with glassine bags in order to evaluate the total number of seeds produced (both retained and shattered). Seed loss (shattered seeds) was estimated as the average difference between the panicles’ weight of the covered plants and those uncovered (i.e., the plants in no-competition situation described above). A correlation between panicles’ weight and the number of panicles was also calculated. This correlation allowed the estimation of the panicles weight in 2014 through counting only the number of panicles.

A destructive sampling was done concurrently with maize harvested in a nearby field: target plants were harvested, different plant parts (stem, leaves, and panicles) were separated and oven dried at 105°C for 36 h, and dry weights were then recorded. In the first year, seeds were separated from rachides and mean dry weight of 100 seeds (in three replicates) was also evaluated to (1) estimate the number of seeds produced by each plant in the different conditions, (2) calculate a correlation between panicle weight and seed weight in order to use the former as indication of reproduction effort (RE) of each phenotype in the different growth conditions (i.e., seed production was estimated using panicle dry weight). The intra-specific competitive ability was evaluated as discussed by Ross and Harper (1972). The space available for plants in no-competitive condition was calculated considering a cube of the radius circle of 1 m (as spaced plants were 2 m apart).

### Statistical Analyses

The dose–response data and ALS enzyme assay data were analyzed using a non-linear regression analysis based on the log-logistic equation (Seefeldt et al., 1995):

$$Y\;\text{=}\;C\;\text{+}\;[(D\;\text{-}\;C)\text{/}[\text{1}\;\text{+}\;{\left(x\text{/}I_{\text{50}}\right)}^{\text{b}}]$$

where *Y* is plant survival or fresh weight, *C* and *D* are the lower and upper asymptotes at high and zero doses, respectively (note: in dose–response data, for biological reasons, and to improve the estimates of the parameters, the upper and lower asymptotes were forced to 100 and 0, respectively), *I*_50_ is the dose giving the 50% response, *b* is the slope and *x* the herbicide rate. For dose–response experiments, doses giving the 50% response, i.e., LD_50_ (based on survival data) and GR_50_ (based on fresh weight data), and relative standard errors, were calculated using the macro BIOASSAY97 developed by Onofri (2005) and running in Windows Excel environment. The resistance index (RI) for the different herbicides was calculated as the ratio between the LD_50_ (GR_50_) of sub-population R and LD_50_ (GR_50_) of sub-population S.

Growth analyses data were analyzed using the software STATISTICA 7 (Hilbe, 2007): linear regression was used to calculate the relation between panicle and seed dry weight and between panicle number and panicle dry weight in experiment 1 in order to estimate the panicle dry weight in experiment 3. To test differences in slopes among regression lines the ANCOVA was performed.

## Results

### Cross-Resistance Pattern of the *E. crus-galli*

The results from the outdoor dose–response pot experiment confirmed the preliminary test conducted by GIRE on a seed sample collected in 2005 from plants that had survived a herbicide treatment with nicosulfuron on the same farm. Plant survival and fresh weight gave similar results (**Table 2**). It was confirmed that S phenotype was adequately controlled by all ALS inhibitors tested, whereas R phenotype was highly cross-resistant to all ALS inhibitors with high RI calculated considering both plant survival and fresh weight (**Table 2**). Apart from nicosulfuron, which controlled more than 90% of R plants when applied at 320 g a.i. ha^-1^ (i.e., 8x), for all other herbicides plant survival was always higher than 50% of the untreated control. Therefore it was impossible to fit the data with the log-logistic equation and LD_50_ values were considered as being higher than the maximum herbicide dose used, e.g., for penoxsulam it was >653 g a.i. ha^-1^ (**Table 2**). For fresh weight, it was not possible to calculate GR_50_ for imazamox: plants were not damaged even at the highest dose applied (640 g a.i. ha^-1^, i.e., 16x). Instead, GR_50_ were calculated for bispyribac-Na and penoxsulam, indicating that plants were damaged, though alive, starting from dose 1x and 4x, respectively. These two herbicides were also more effective on the S phenotype and plants were already damaged at very low doses, therefore the RI resulted as very high (**Table 2**).

**Table 2:** Parameters of the log-logistic equations for whole plant dose–response of sub-population S and R, for the four herbicides tested; LD_50_ and GR_50_ are the herbicide doses causing 50% reduction in plant survival and fresh weight, respectively; standard errors are given in brackets.

Herbicide	Sub-population	*b* (slope)	LD_50_ (g a.i. ha^-1^)	Residual sum of squares	RI^a^
**Survival**					
Nicosulfuron	S	4.7 (0.4)	20 (0.6)	505	
	R	21 (1.9)	383 (30)	306	19
Penoxsulam	S	3.6 (0.9)	12 (1.0)	4,459	
	R	–	>653	–	>56
Bispyribac-Na	S	6.5 (0.9)	5.6 (0.3)	731	
	R	–	>490	–	>42
Imazamox	S	4.4 (0.8)	7.5 (0.4)	1671	
	R	–	>640	–	>85

**Herbicide**	**Sub-population**	***b* (slope)**	**GR_50_ (g a.i. ha^-1^)**	**Residual sum of squares**	**RI^a^**

**Fresh weight**					
Nicosulfuron	S	6.6 (0.9)	21 (1.1)	871	
	R	5.4 (0.9)	361 (9.3)	361	17
Penoxsulam	S	2.9 (0.7)	5.9 (0.6)	4,150	
	R	1.6 (0.2)	330 (26)	1,022	56
Bispyribac-Na	S	1.6 (0.1)	1.7 (0.1)	307	
	R	1.1 (0.1)	56 (6.6)	2,167	32
Imazamox	S	2.8 (0.4)	4.9 (0.2)	445	
	R	–	>640	–	>132

*^a^RI, resistance indexes calculated as the ratio of LD_*50*_ R over LD_*50*_ S or GR_*50*_ R over GR_*50*_ S.*

### Resistance Mechanisms

Molecular analyses proved that a target-site resistance mechanism is involved. The PCR amplification with ECH-ALS-F and ECH-3R led to obtaining a 1,870 bp sequence including all the known conserved domains identified previously carrying mutations endowing resistance. In all the resistant plants a double nucleotide substitution GC-AA, giving an Ala-Asn change at amino acid position 122 of the ALS gene, was detected. All plants were heterozygous for the mutation at the ALS locus. In the susceptible plants not one known mutation was identified.

**FIGURE 1:**
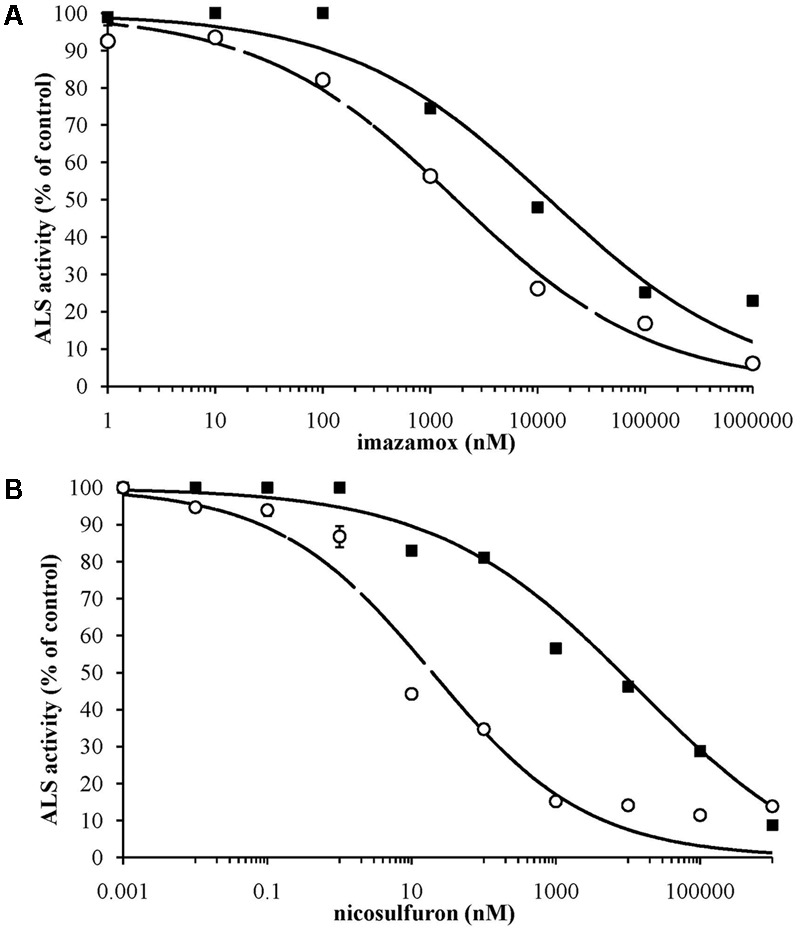
Inhibition curves by imazamox **(A)** and nicosulfuron **(B)** of ALS enzyme extracted from S (– –○– –) and R (–■–) sub-populations. Curves represent the response predicted by non-linear regression; symbols represent percentage of mean ALS activity, based on the untreated controls. Standard errors are reported as vertical bars.

The *in vitro* measurements of ALS activity indicated a sigmoidal response to increasing doses of imazamox and nicosulfuron (**Figure 1**) and the data were well fitted by the log-logistic equation (**Table 3**). The results of the *in vitro* bioassay support the presence of a target-site mediated as main resistance mechanism. The R/S ratios calculated were 7.4 for imazamox and 413 for nicosulfuron. The large difference in *I*_50_ between R (*I*_50_ = 7980) and S (*I*_50_ = 19) sub-populations led to the exclusion of non-target-site mediated resistance mechanisms for the selecting agent of the resistant phenotype, i.e., nicosulfuron. This is in keeping with the results obtained from the molecular analyses.

**Table 3:** Parameters of the log-logistic equations for ALS enzyme activity of sub-population S and R, for the two herbicides tested; *I*_50_ is the herbicide dose causing 50% reduction in ALS activity; standard errors are given in brackets.

Herbicide	Sub-population	*b* (slope)	*I*_50_ (nM)	Residual sum of squares	R/S ratio^a^
Nicosulfuron	S	0.4 (0.1)	19 (9.4)	555	
	R	0.3 (0.1)	7,980 (930)	146	413
Imazamox	S	0.5 (0.0)	1,735 (274)	68	
	R	0.5 (0.1)	12,830 (4,075)	263	7

*^a^R/S ratio, resistance indexes calculated as the ratio of *I*_*50*_ R over *I*_*50*_ S.*

### Comparative Growth Analyses

All *E. crus-galli* seedlings used as target plants were analyzed to verify the presence or not of the mutant ALS allele. All resistant plants had the double substitution GC-AA, giving an Ala-122-Asn amino-acid change, whereas none of the susceptible plants had any nucleotide substitution.

**FIGURE 2:**
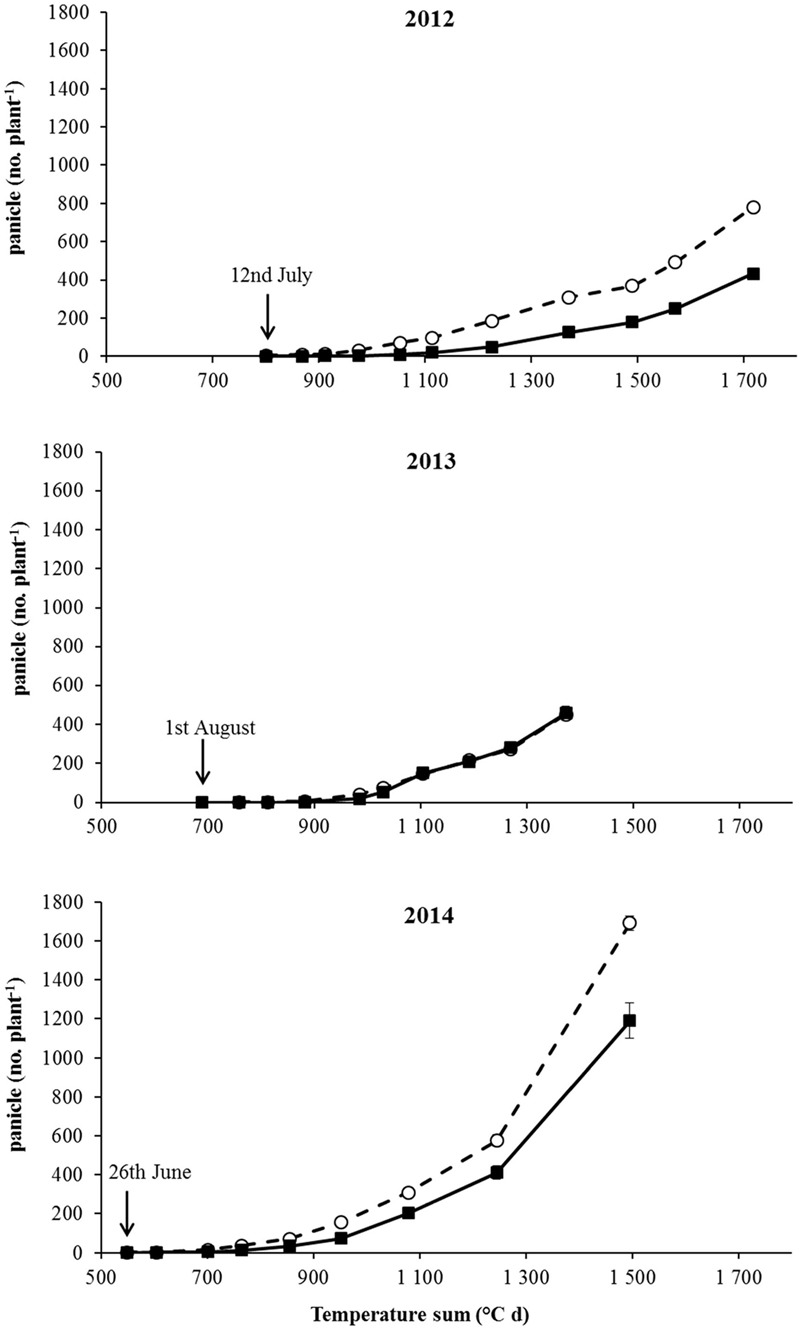
Panicle number under no-intraspecific competition from the beginning of flowering to harvest for S (– –○– –) and R (–■–) plants in the three experiments (2012, 2013, and 2014). The arrows report the dates of the flowering start. Vertical bars represent standard errors.

**FIGURE 3:**
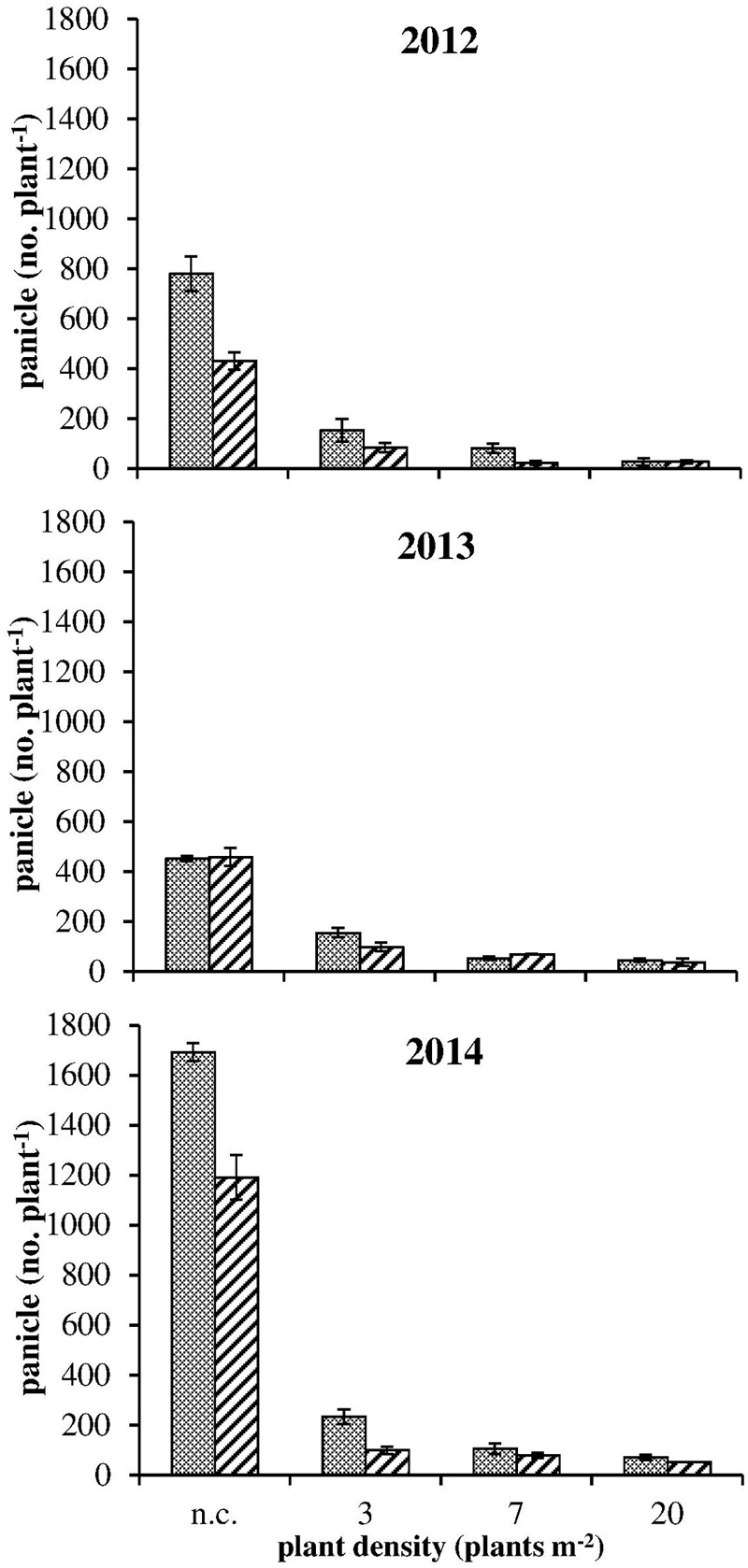
Panicle number at harvest for S 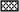 and R 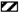 plants in competition (3, 7, and 20 plants m^-2^) and in no-competition (n.c.) condition in the three experiments. Vertical bars represent standard errors.

The onset of flowering was delayed of about 1 month in 2013 in comparison to 2012 and 2014 (**Table 4**). In all years a later flowering in R plants with respect to S ones was recorded in both competitive and non-competitive situations (**Table 4**). Considering the cumulative number of panicles under no-competition in relation to the temperature sum (**Figure 2**), the same delay of about 1 month was recorded in 2013. It is worth mentioning that the temperature sum at the end of August 2013 was around 1,000°C d, whereas this value was reached at the beginning of August in 2012 and 2014. The production of panicles for R and S plants in 2013 was similar, whereas at harvest R plants produced 55% and 70% less panicles than S plants in 2012 and 2014, respectively (**Figure 2**). Considering the intraspecific competition, the larger differences between R and S plants were recorded in 2013 and 2014 at the lowest density (3 plants m^-2^) and progressively disappeared at higher plant densities (**Figure 3**). No significant differences were detected in 2012, likely due to the higher variability in panicles produced by S plants.

**Table 4:** Beginning of flowering of the S and R target plants in no-intraspecific competition and competition (3, 7, and 20 plants m^-2^) in the three experiments.

		No competition	Competition (plants m^-2^)		
			3	7	20
2012	S	10-July	12-July	12-July	25-July
	R	17-July	20-July	30-July	30-July
	Δ (d)	7	8	8	5
	Δ (°C d)	99	110	252	78
2013	S	5-August	1-August	5-August	1-August
	R	13-August	8-August	13-August	13-August
	Δ (d)	8	7	8	12
	Δ (°C d)	124	123	124	194
2014	S	26-June	29-June	29-June	1-July
	R	29-June	1-July	9-July	9-July
	Δ (d)	3	2	10	8
	Δ (°C d)	36	56	116	97

*The difference (Δ) between S and R phenotypes is reported in days (d) and temperature sum (°C d).*

Evaluation of the dry biomass, in particular the panicle dry weight produced by S and R plants, consolidated the data from the phenological analyses. The highly significant correlation (*R*^2^ = 0.99) between seed dry weight (separated from rachides) and panicle dry weight in the first year of experiment allowed this latter variable to be used to estimate the seed production of S and R plants. In 2012 and 2013, panicle number and panicle dry weight were recorded for R and S plants and a linear regression analysis between these two parameters was obtained. In 2012, the *R*^2^ was 0.98 (linear equation: *y* = 0.675*x*), whereas in 2013 the *R*^2^ was slightly lower (0.93). Bartlett’s test for homogeneity of variance revealed that it was not possible to analyze all data together. Therefore, because of the higher *R*^2^ only 2012 data were used for the estimation of panicle dry weight in the last experiment.

**FIGURE 4:**
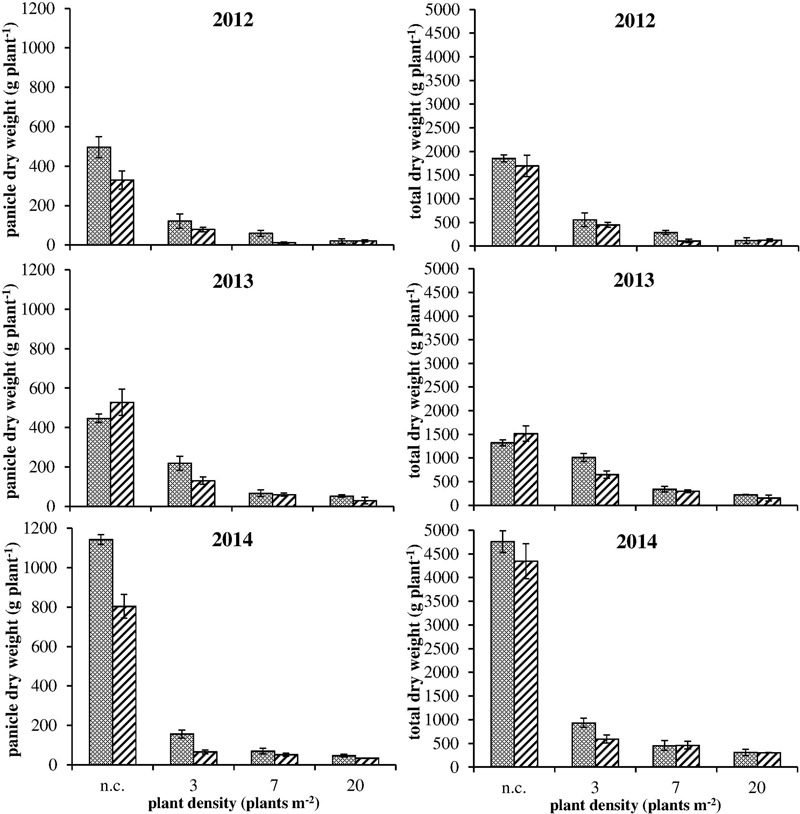
Panicle dry weight (on the left) and total dry weight (on the right) for S 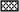 and R 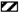 plants under intraspecific competition (3, 7, and 20 plants m^-2^) and no-competition (n.c.) in the three experiments. Vertical bars represent standard errors.

**FIGURE 5:**
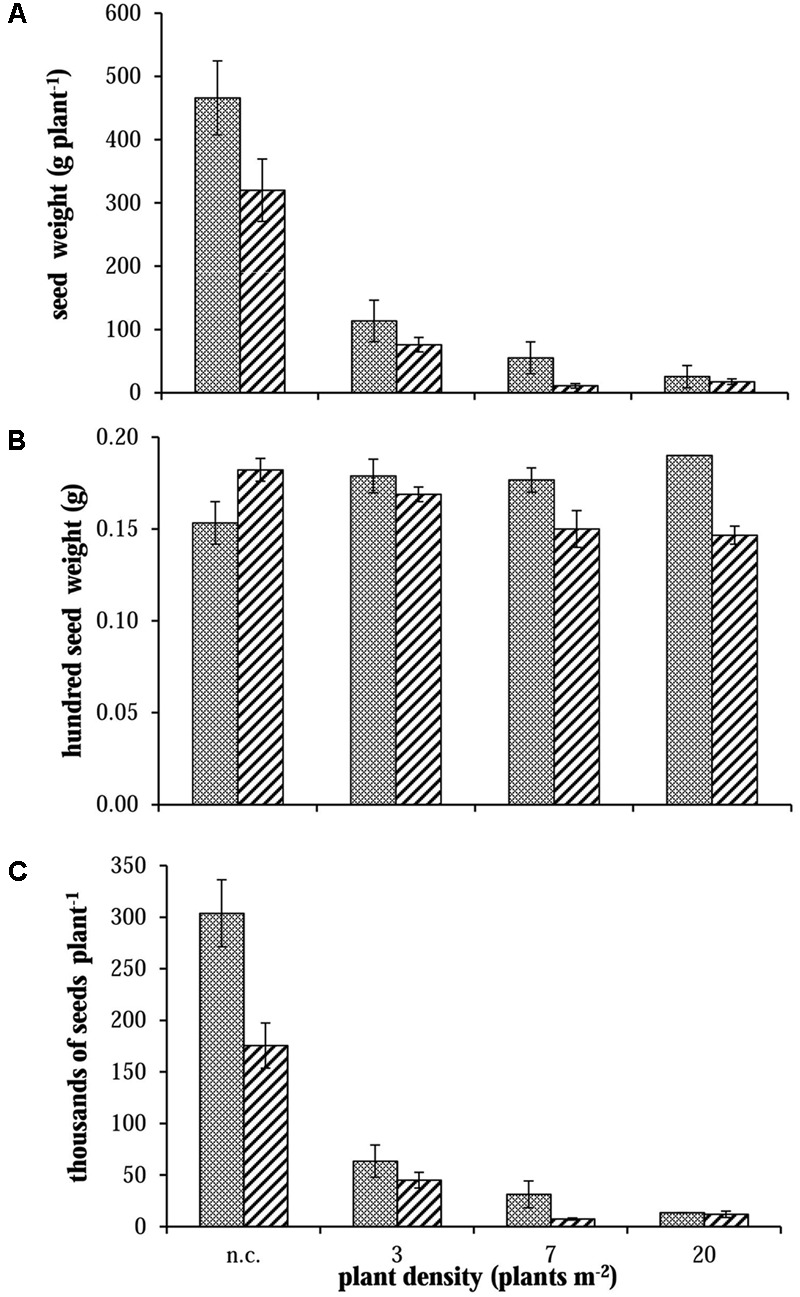
Evaluation of **(A)** seed dry weight, **(B)** mean weight for 100 seeds and **(C)** seed number produced per plant for S 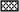 and R 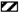 phenotypes in 2012 in no-competition (n.c.) and competition (3, 7, and 20 plants m^-2^). Vertical bars represent standard errors.

The comparison between different vegetative plant parts (stems and leaves) did not highlight any difference between R and S plants (data not shown). Considering the total dry weight (**Figure 4**, on the right): a difference was detected in only two cases (i.e., 2012 – 7 plants m^-2^ and 2013 – 3 plants m^-2^). Instead, considering panicle dry weight, S plants allocated more biomass to the reproductive organs than R ones especially in no-competition and at lower plant densities (**Figure 4**, on the left). At the highest plant density (i.e., 20 plants m^-2^) the competition among plants was high and panicle production very low, giving no differences in reproductive effort between S and R plants. Data from 2012 and 2014 were consistent, whereas in 2013 a slight difference (not significant) in panicle dry weight between S and R plants was detected only in the 3 plants m^-2^ situation (**Figure 4**). Other differences between S and R phenotypes were detected analyzing seed weight in 2012. Seed dry weight of S plants was about 30% higher than in R plants in no-competition situation (**Figure 5A**). Also in this case, the difference decreased enhancing the competition level. However, the mean weights of 100 seeds evaluated for the different situations have a different trend: mean seed weight increased with competition level for S plants, whereas it decreased for R ones (**Figure 5B**). In no-competition situation, the mean seed weight of S plants was lower than that of R ones, but S plants produced much more biomass. The resulting estimated number of seeds produced by R and S plants was significantly different (**Figure 5C**), i.e., the lower weight of a single seed in the S plant increases the difference in the number of seeds between S and R plants. The S plant produced 43% more seeds than the R one (**Figure 5C**). This difference disappeared at the highest plant density.

**FIGURE 6:**
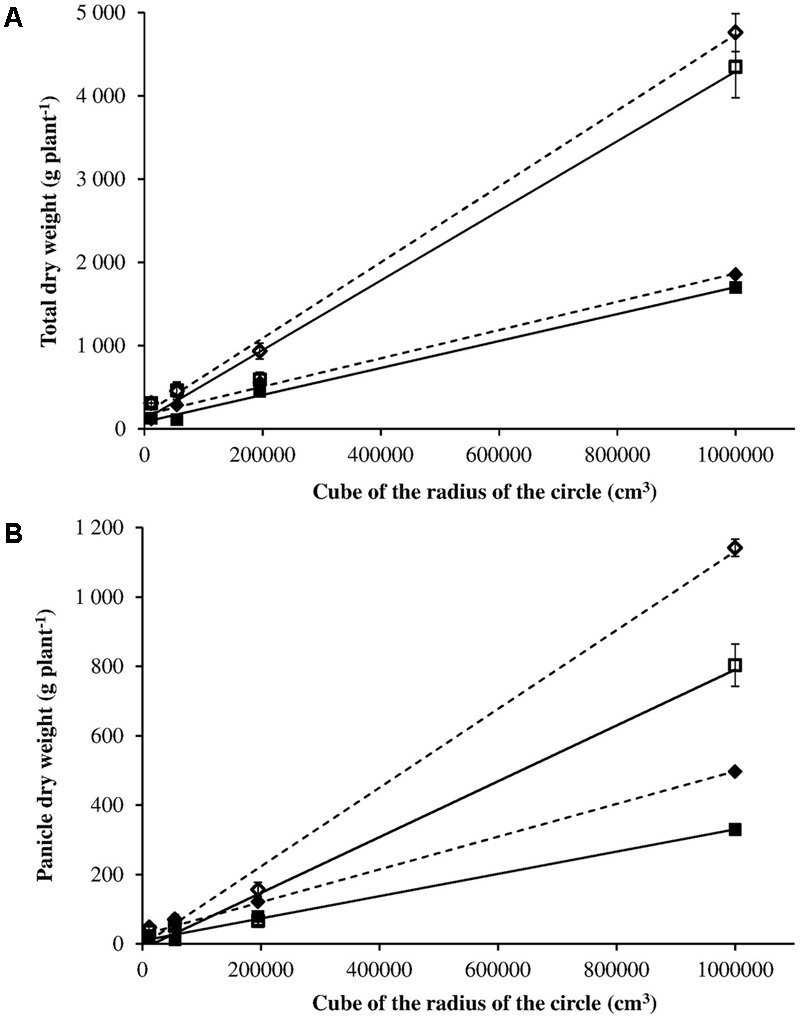
Panicle dry weight **(A)** and total dry weight **(B)** in relation to the three-dimensional space that plants have available expressed as cube of radius of the circle for S (– – –) and R (–) phenotypes in 2012 (◆ ■) and 2014 (◇ □).

In order to compare the intra-specific competitive ability (i.e., between R and S plants), the relation between total dry weight (or panicle dry weight) and the three-dimensional space that plants have available, expressed as cube of radius of the circle, was calculated (**Figure 6**) (Ross and Harper, 1972). Considering the total dry weight, the slope of the regression lines between S and R plants was not significantly different in both 2012 and 2014 (*t*-value = 0.719, *P* = 0.512 in 2012; *t*-value = 1.02, *P* = 0.366 in 2014) (**Figure 6A**). Different behavior is observed for panicle dry weight (**Figure 6B**). The slopes of the regression lines between S and R plants were significantly different in both 2012 and 2014 (*t*-value 7.410, *P* = 0.002 in 2012; *t*-value 3.209, *P* = 0.03 in 2014). The more space available for plant growth the bigger the difference is in the biomass allocated to panicles (and therefore to seeds) between R and S plants. This implies that R plants have a lower potential for seed production, in other words they suffer from a fitness penalty in comparison with S plants.

## Discussion

### ALS Gene Sequencing Revealed a Novel Ala-122-Asn Substitution

This study reports for the first time the Ala-122-Asn amino-acid change in the ALS gene that confers a high level of cross-resistance to ALS inhibitors worldwide. Resistance-endowing mutations at Ala-122 have so far been reported in seven weed species (Tranel et al., 2017). The Ala-122-Thr endowed resistance only to imidazolinones but no resistance to sulfonylureas and pyrimidinylthiobenzoates in *Solanum ptycanthum, Amaranthus retroflexus, A. powellii*, and *Apera spica-venti* (Tranel et al., 2017). In *E. crus-galli* from Arkansas, this amino acid change conferred resistance not only to imidazolinones but also to triazolopyrimidines (Riar et al., 2013). The Ala-122-Val did not confer a broad resistance to the ALS inhibitors either (Riar et al., 2013; Tranel et al., 2017). Instead, the double point mutation GCT-TAT that determines an Ala-Tyr amino-acid change, detected only in *R. raphanistrum* (Han et al., 2012) conferred high-level and broad-spectrum resistance to ALS-inhibiting herbicides (Han et al., 2012; Tranel et al., 2017). Similarly, in *E. crus-galli* populations analyzed herein, the Ala-122-Asn amino-acid change in the ALS gene confers a broad spectrum resistance. Overall, resistance-endowing mutations at Ala-122 of the ALS gene can determine a different resistance pattern according to weed species and amino acid change. This reinforces the idea that each resistance case needs a specific study.

### Evidence of Fitness Costs in Ala-122-Mutated Plants

The resistance costs associated to specific ALS resistance-endowing mutations that have evolved in field weed populations have been examined in a limited number of cases. Here, resistant and susceptible plants of *E. crus-galli* with similar genetic background were selected and the detection of a target-site mediated resistance mechanism involved has allowed fitness costs associated with resistance to ALS herbicides to be estimated and interpreted.

Results of the 3-year growth analysis indicate that a fitness cost is present in plants bearing the Ala-122-Asn substitution. Differences were observed in growth and development: a slower development of R phenotype in relation to the S one was recorded (**Table 3**). Even if during the second year experiment, the date of beginning of flowering was delayed by 1 month with respect to 2012 and 2014, this 1 month delay was recorded throughout the life cycle, from appearance of the first panicle until harvest time (**Table 3**). As previously argued, the results of field experiments can hardly be treated quantitatively because many indeterminate factors, including the weather, may be of great importance (De Wit, 1960). However, our results highlight that the differences between R and S plants were consistent in different years and climatic conditions. Furthermore, in no-competition condition, R plants produced about 40% fewer panicles than S plants (**Figure 2**) and the high panicle dry weights recorded for S plants indicate that they allocated more biomass to reproductive organs than the R ones. It can be inferred that the lack of differences between R and S in the number of panicles produced in 2013 can be attributed to the different climatic condition. During 2013, there was a rather rainy spring with respect to 2012 and 2014 that delayed seedling emergence and therefore the subsequent phenological growth phases.

Considering the competitiveness of R and S phenotypes, the results indicated that the higher the plant density the less the difference was between R and S phenotypes in terms of number of panicles produced. No differences were recorded at the highest plant density. Hence, at low plant densities the susceptible plants exhibited a potential fitness advantage. The only study of fitness costs on mutation at Ala-122 was conducted on *R. Raphanistrum* having the Ala-122-Tyr mutation and no evidence of pleiotropic effects on plant growth were recorded between R and S plants (Li et al., 2013).

Overall, it seems that fitness costs related to ALS resistance vary according to the species, the mutated allele conferring resistance, the genetic background, experimental conditions (glass-house, field) and environmental conditions involved. This study reveals that fitness costs associated to Ala-122-Asn mutant *E. crus-*galli plants are evident under good growth conditions, but progressively disappear in highly competitive conditions.

### Management/Evolution of ALS-Resistant *E. crus-galli*

Experiments conducted under field conditions allow fitness to be compared in a real situation where resistance evolves and has to be managed. This study proves that the evolved resistance to ALS inhibitors in *E. crus-galli* due to the Ala-122-Asn amino-acid change is associated with a fitness cost. However, the impact of this penalty in the absence of ALS inhibitors may be influenced by many factors, including environmental conditions (Gassmann, 2005). Different climatic conditions during 2013 influenced the relative plant growth, reducing the disadvantages of R plants toward the S plants.

The differential population dynamics observed between R and S *E. crus-galli* phenotypes can be a good starting point for devising a resistance management strategy. Differences in life history traits can be manipulated in fields to minimize or reverse the resistance evolution (Vila-Aiub et al., 2011). In our case the lack of a fitness cost at high plant densities suggests that keeping the infestation density as low as possible can increase the reproductive success of the S phenotype and therefore contribute to lowering the ratio between R and S alleles. In a situation where ALS-resistance is well established, a longer-term management based on crop rotation, lack of selection pressure from ALS-inhibiting herbicides and the use of any other available control tool should help to induce some change in the R/S plant ratio.

The different weather conditions encountered in 2013 may suggest other management options. The frequent and relatively abundant rainfall events in spring delayed maize sowing and weed control operations for about 1 month in relation to the normal schedule. As a consequence, the emergence of *E. crus-galli*, usually characterized by two main fluxes of emergences regulated by rainfall and temperature, was instead only one large and longer flux. In this case, the delay in sowing helped the control of weeds.

Another experiment was conducted in the same maize field where the conventional weed control strategy (with ALS inhibitors) adopted by the farm was compared with another one that did not include these herbicides for 4 years. The results showed that the simple measure of ceasing to use the selecting agent does not significantly modify the ratio between R and S plants when the barnyardgrass seed bank initially had more than 95% of “R seeds” (Scarabel et al., unpublished data). It is clear that the long-lived seed bank of *E. crus-galli* (Holm et al., 1977) imposes long-term resistance management strategies when the seed bank contains predominantly “R seeds.”

It is widely accepted that herbicides are not silver bullets (e.g., Mortensen et al., 2000) for the management of resistance, but rather diversified control tools should be used as “little hammers” (Barzman et al., 2015). If adequately embedded in a medium or long-term integrated weed management strategy, the presence of R plants with a fitness penalty provides an opportunity to minimize or reverse herbicide resistance evolution.

## Author Contributions

SP, MS, and LS designed the experiments. SP and VR carried out the field and laboratory experiments. SP, LS, and MS analyzed the data and wrote the manuscript. MS supervised this study. All authors read and approved the manuscript.

## Conflict of Interest Statement

The authors declare that the research was conducted in the absence of any commercial or financial relationships that could be construed as a potential conflict of interest.

## Footnotes

1 www.arpa.veneto.it
