# Supplementary figures and images for: A New Ala-122-Asn Amino Acid Change Confers Decreased Fitness to ALS-Resistant Echinochloa crus-galli
Source: Front Plant Sci. 2017 Nov 28;8:2042. doi: 10.3389/fpls.2017.02042 (PMC5712356; doi:10.3389/fpls.2017.02042)

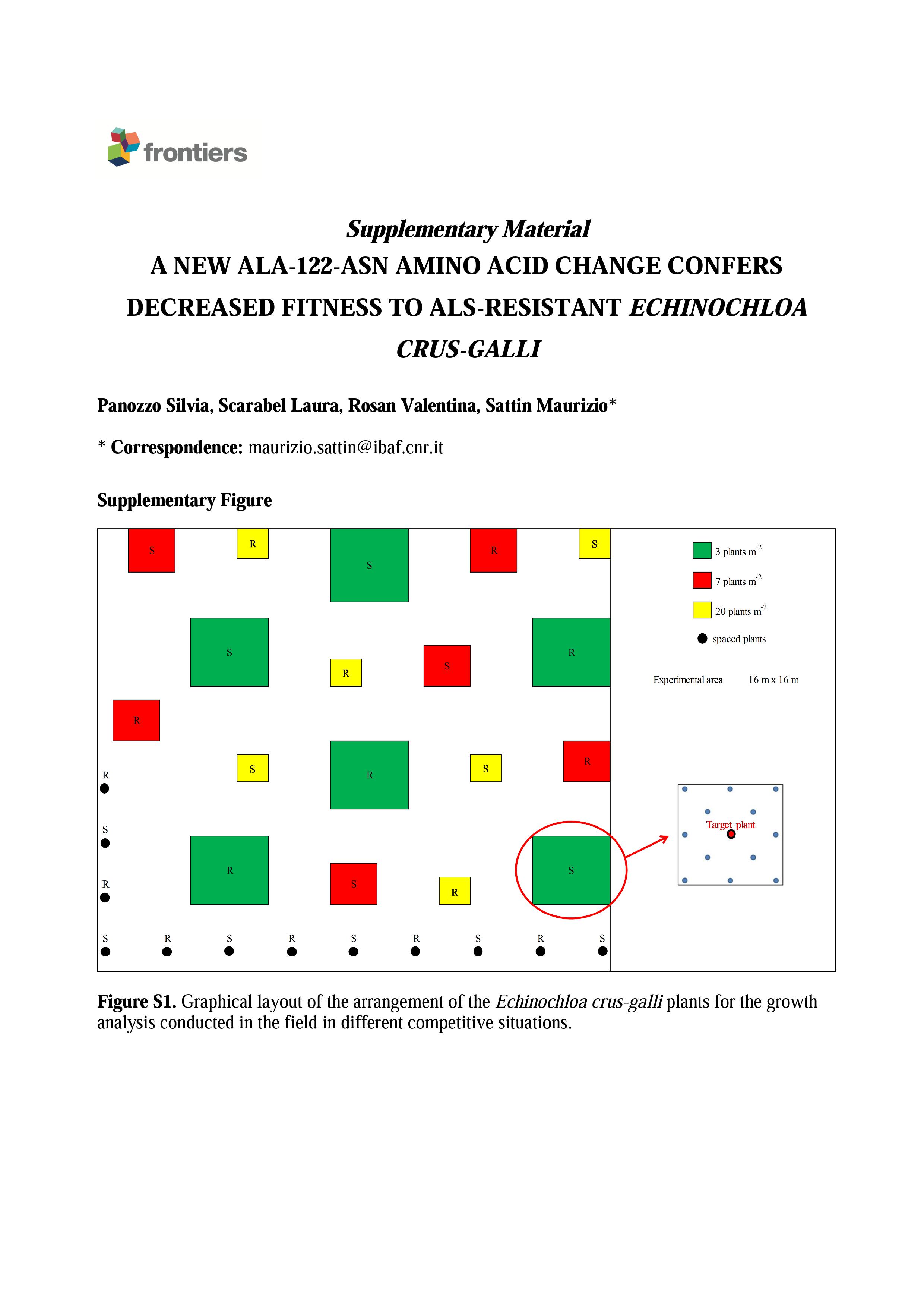

Supplement: Supplementary file 1 [file Image_1.JPEG]
